# A high-density microelectrode-tissue-microelectrode sandwich platform for application of retinal circuit study

**DOI:** 10.1186/s12938-015-0106-5

**Published:** 2015-11-26

**Authors:** Frank Yang, Chung-Hua Yang, Fu-Min Wang, Ya-Ting Cheng, Chih-Ciao Teng, Li-Jen Lee, Chang-Hao Yang, Long-Sheng Fan

**Affiliations:** Institute of NanoEngineering and Microsystems, National Tsing-Hua University, Hsin-Chu, Taiwan; Graduated Institute of Anatomy and Cell Biology, National Taiwan University, Taipei, Taiwan; Department of Ophthalmology, National Taiwan University Hospital, Taipei, Taiwan

**Keywords:** Retina, Micro-electrode array (MEA), Retinal prosthesis, Oxygen consumption, Retinal ganglion cells, Firing rate, Neural circuit

## Abstract

**Background:**

Microelectrode array (MEA) devices are frequently used in neural circuit studies, especially in retinal prosthesis. For a high throughput stimulation and recording paradigm, it is desirable to obtain the responses of multiple surface RGCs initiated from the electrical signals delivered to multiple photoreceptor cells. This can be achieved by an high density MEA-tissue-MEA (MTM) sandwich configuration. However, the retina is one of the most metabolically active tissues, consumes oxygen as rapidly as the brain. The major concern of the MTM configuration is the supply of oxygen.

**Methods:**

We aimed to develop a high density MTM sandwich platform which consists of stacks of a stimulation MEA, retinal tissue and a recording MEA. Retina is a metabolically active tissue and the firing rate is very sensitive to oxygen level. We designed, simulated and microfabricated porous high density MEAs and an adjustable perfusion system that electrical signals can be delivered to and recorded from the clipped retinal tissue.

**Results:**

The porous high-density MEAs linked with stimulation or recording devices within a perfusion system were manufactured and the MTM platform was assembled with a retina slice inside. The firing rate remained constant between 25 and 55 min before dramatically declined, indicating that within certain period of time (e.g. 30 min after habituation), the retina condition was kept by sufficient oxygen supply via the perfusion holes in the MEAs provided by the double perfusion system.

**Conclusions:**

MTM sandwich structure is an efficient platform to study the retinal neural circuit. The material and arrangement of high density microelectrodes with porous design make this MEA appropriate for sub-retina prosthesis. Finding ways to prolong the recording time and reduce the signal-to-noise ratio are important to improve our MTM prototype.

## Background

In outer retinal degenerative diseases, such as the age-related macular degeneration (AMD) and hereditary Retinitis Pigmentosa (RP), although photoreceptors are lost, numerous bipolar and retinal ganglion cells (RGCs) are still preserved [1, 2]. Retinal prosthetic devices are being developed to bypass degenerated photoreceptors by directly or indirectly activating the surviving retinal neurons with electrical stimulation [3], which might improve vision in patients with retinal degenerative diseases. While these developments are very encouraging, many impediments still remain, for example, the electrophysiological processes that take place within the retina during the stimulation from these retinal prostheses are still not fully characterized. The actuality of visual information in the RGCs is hard to be reproduced by the electrical signals delivered to the photoreceptors or bipolar cells due to the complicated cellular cascade in the retina [4–6].

While RGCs are activated by electrical signals delivered to photoreceptors, point-to-point responses are key to decode the retinal information process. Various in vitro models are developed to solve the puzzle [7–16]. Microelectrode arrays (MEAs) are being used for either recording multiple RGCs or stimulating numerous photoreceptors at different sites. However, for a high throughput stimulation and recording paradigm, it is desirable to obtain the responses of multiple surface RGCs initiated from the electrical signals delivered to multiple photoreceptor cells. This can be achieved by an MEA-tissue-MEA (MTM) sandwich configuration [17].

The major concern of the MTM configuration is the supply of oxygen. The retina is one of the most metabolically active tissues, consumes oxygen as rapidly as the brain [18–21]. The retina has a dual circulation system: the photoreceptors and the greater portion of outer plexiform layer receive oxygen from the choriocapillaris whereas the inner retinal layers are supplied by the superficial and deep capillary plexuses formed by branches of the central artery of retina [22–25]. The structural and functional integrity of the retina depends on a regular oxygen supply. The physiological condition of RGCs is particularly sensitive to hypoxic stress. Under hypoxic conditions, the firing rate of RGCs is reduced [26]. For example, in cat retina, both X and Y RGCs exhibit constant firing rates, independent of location, if the arterial oxygen tension (PO_2_) level is greater than 45 mmHg. While arterial PO_2_ values are between 24 and 34 mmHg, the RGC firing rates are increased and then followed by a decrease. If the arterial PO_2_ value is below 24 mmHg, the RGCs show a train of large firings but a complete cessation afterward within 5 min. Other experiments also indicate that the electrical activity of inner retina is unaffected during systemic hypoxia as long as the arterial PO_2_ is above 40 mmHg [27]. Normally, the PO_2_ inside of retina is lower than the arterial PO_2_. The minimum PO_2_ at the retina is about 17.4 mmHg in dark adapted in the Long-Evans rat retina measured with oxygen-sensitive microelectrodes. Mammals such as cat, Long-Evans rat, mice, and macaque has similar oxygen consumption behavior by the retina [27–32]. The condition of Long-Evans was used as an example in this study. Since retinal oxygen consumption rate in dark adaption is higher than in light adaption, this value, 17.4 mmHg, is therefore considered as the minimum retinal PO_2_ required for maintaining the normal response of RGCs. For an MTM platform, if the PO_2_ within the clipped retinal tissue exceeded the minimal PO_2_ requirement and was maintained, it could be a useful tool to study the retinal circuits.

Here we report the design, microfabrication and test of an MTM sandwich platform for retinal neural circuit study. The problem of oxygen supply was overcome by the opening of holes in the middle of the high-density MEAs on the top of and below the retina slice. The hole-opening ratio and flow speed of oxygenated fluid were concerned and computer simulation was used to ensure that the minimum PO_2_ within the retina was exceeded. The distribution of the perfusion pores was also considered to accommodate the size and pitch of the microelectrodes of the MEAs to achieve the high-density resolution which is critical for prosthesis of high visual acuity [33].

## Methods

**Fig. 1:**
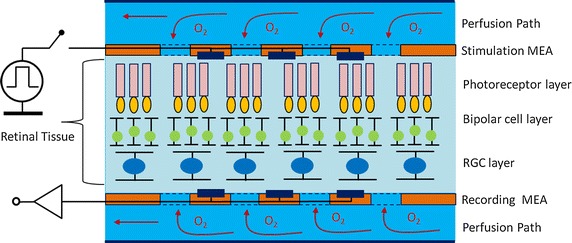
An ideal MEA-Tissue-MEA (MTM) platform. It illustrates the perfusion paths of oxygenized fluid, stimulation MEA, recording MEA and retina tissue. Both stimulation and recording MEAs have porous holes to improve the oxygen diffusion rate

To maintain the oxygen supply to the retina tissue within the MEA-tissue-MEA (MTM) sandwich structure, we designed perforated MEAs with uniformly distributed diffusion holes (Fig. 1). We assumed that the oxygen diffusion effect dominates the oxygen transfer in the retina-media interface in the MTM structure. Using Fick’s law, the oxygen diffusion flux through the interface is

1 $${\text{J}} = {\text{D}}*{\text{k}}*\left( {\frac{\text{dp}}{\text{dz}}} \right)$$

where J is flux of oxygen diffusion, D is the oxygen diffusion constant, k is the solubility coefficients, and D * k is 2.84 × 10^−6^ ml cm^2^/(100 g min mmHg) [31]. P is the oxygen tension and z is the direction along thickness of retina. We evaluated the amount of oxygen tension needed for sufficient retinal tissues oxygenation under the condition of a 18.9 % open area ratio (OAR, ratio of diffusion holes area relative to the MEA surface area) by assuming an equilibrium of the total retinal oxygen consumption to the total flux through the interface. 18.9 % is the average value of whole diffusions of the MEA surface, limited by process capability:

2 $$\int\limits_{\text{Volume of retina}} {{\text{Q}}_{\text{NR}} \cdot {\text{dV}}} = \int\limits_{{{\text{Area of }}2 {\text{MEA surfaces}}}} {{\text{J}} \cdot {\text{dA}}}$$

then

3 $${\text{Q}}_{\text{NR}} \times {\text{A}} \times {\text{retina}}\;{\text{thickness}} = {\text{D}} \times {\text{k}} \times \left( {\frac{\text{dP}}{\text{dz}}} \right) \times 2{\text{A}} \times {\text{OAR}}$$

The oxygen distribution and consumption in the retina are similar for several mammals (Long-Evans rat, cat, monkey and albino rat). The oxygen consumption rate of a normal retina in dark-adapted (Q_NR_) is ~3.5 ${\text{ml O}}_{2} /\left( {100\,{\text{g}}\;{ \hbox{min} }} \right)$ [27–32] was used in the calculation.

**Fig. 2:**
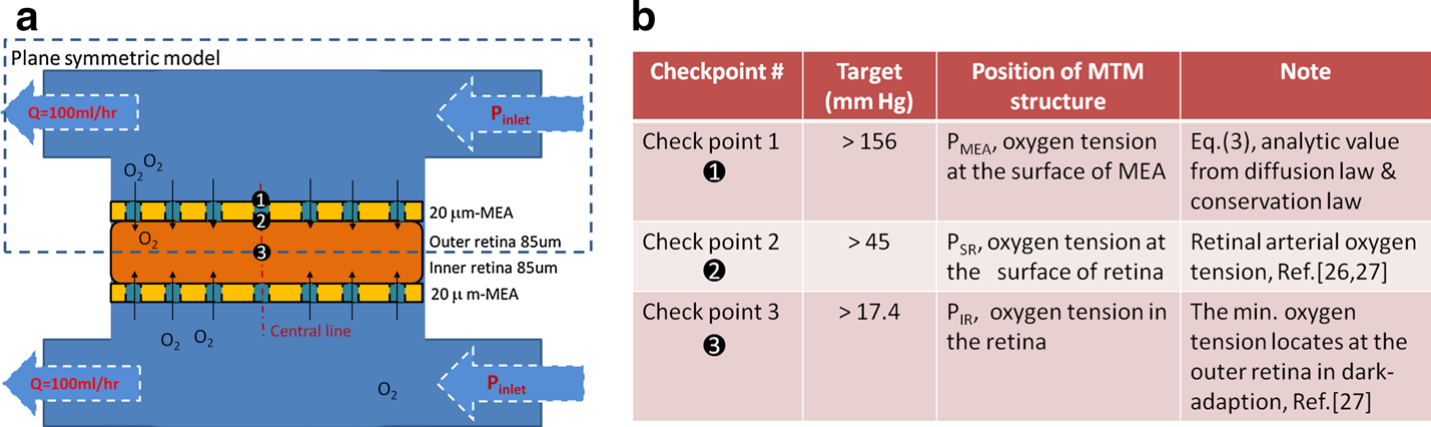
Physical model and physical parameters of MTM. **a** A model of MTM platform labeled with predetermined parameters and the positions of three checks points. The perfusion paths could improve oxygen transfer rate and also create negative fluidic pressure to properly put retina tissue on MEAs with minimum gaps. **b** The required PO_2_ in the three check points are listed

The inner and outer retina are oxygenated by two separated perfusion systems, and we assumed the average thicknesses of the inner retina and the outer retina are both 85μm in thickness for a 170 μm-thick retina [34] and the thickness of MEA was set 20 μm (Fig. 2). We then approximated

4 $${\text{dP}}/{\text{dz}}\sim \frac{{{\text{P}}_{\text{SMEA}} - {\text{P}}_{\text{SR}} }}{{(20 { \mu}{\text{ m}})}}$$

where P_SR_ is the oxygen tension at the surface of the retina (check point 2 in Fig. 2) which was set to be the arterial oxygen tension, 45 mmHg [27], P_SMEA_, the oxygen tension at the surface of MEA (check point 1 in Fig. 2), would be 156 mmHg that is possibly achieved in hyperoxic medium. However, the oxygen tension in the fluid chamber is a function of flow rate, concentration gradient, and time. We set the flow rate 100 ml/h and calculated the oxygen distribution of the inner retina. The minimum PO_2_ within the retina (check point 3 in Fig. 2), P_IR_, 17.4 mmHg [27], was also been considered in the model.

The transport (fluid convection and gas diffusion) and reaction equations were considered with the Newton-laminar flow to build the governing equation.

5 $$\frac{{\partial {\text{P}}}}{{\partial {\text{t}}}} + {\text{u}} \cdot \nabla {\text{P}} = 2 \times {\text{D}}*{\text{k}}_{\text{H}} \left( {\frac{{{\text{d}}^{ 2} {\text{P}}}}{{{\text{dx}}^{2} }}} \right) + {\text{Q}}_{\text{NR}} ,$$

The distribution of oxygen tension in the fluid was simulated using Finite Element Method (FEM) software (COMSOL Multiphysics, COMSOL, Inc, USA).

### Measurement of oxygen concentration

The concentration of oxygen in the medium was measured using a dissolved oxygen meter (Lutron DO-5509) and converted to the oxygen tension (P) using Henry Law

6 $${\text{k}}_{\text{L}} = \frac{{{\text{c}}_{\text{a}} }}{\text{p}}$$

where c_a_ is concentration of oxygen in the medium, ${\text{k}}_{\text{H}} \left( {37\,^\circ {\text{C}}} \right) = 3.09 \times 10^{ - 5} {\text{mg}}/\left( {\text{ml mmHg}} \right)$ and the value of P (oxygen tension) could be calculated.

### Fabrication process

**Fig. 3:**
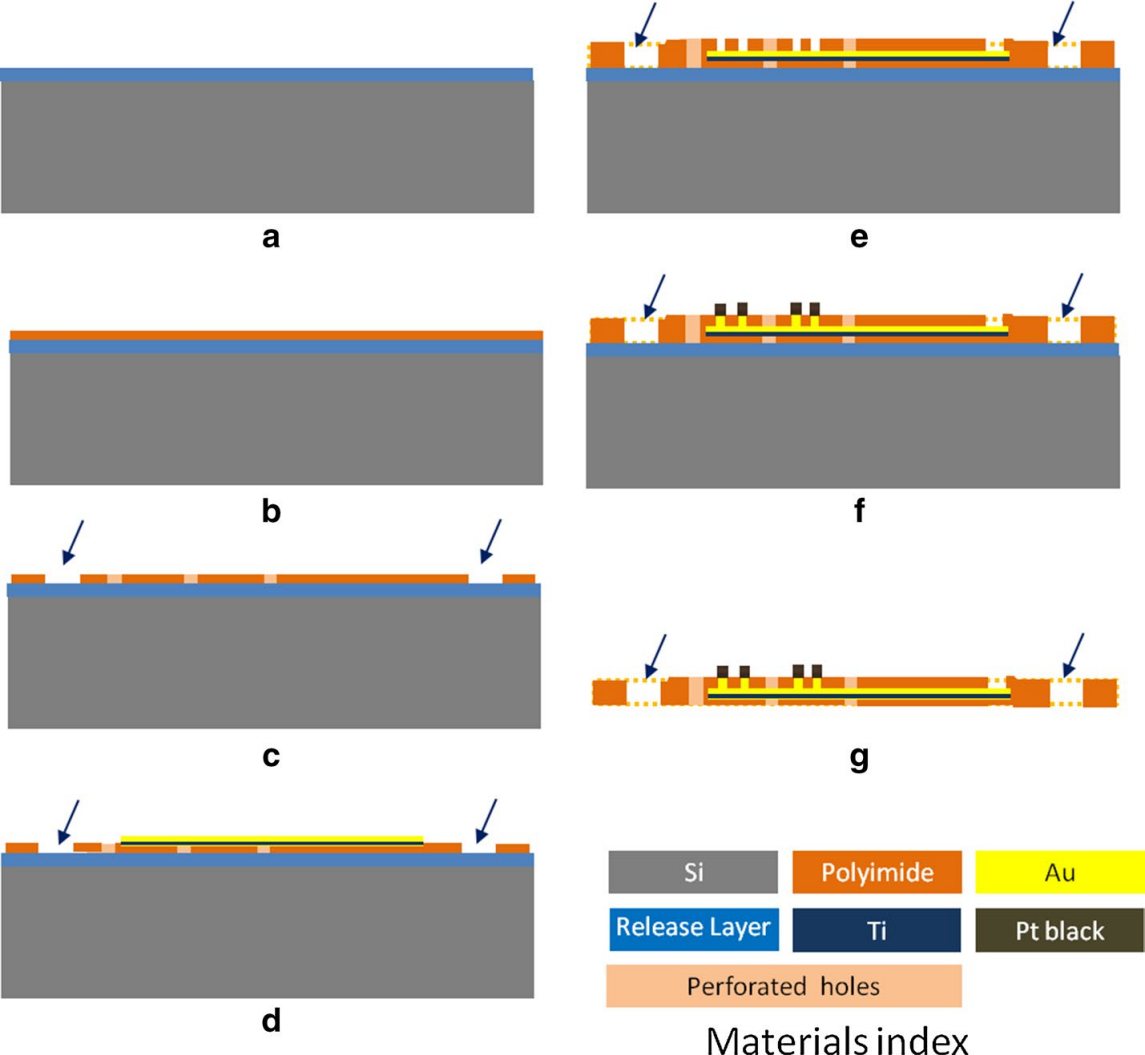
The fabrication process of perforated MEA: **a** substrate with release layer deposited; **b** first polyimide spin coating; **c** polyimide patterning and curing; **d** Metal layer (Au and Ti) deposition and patterning; **e** second polyimide coating, patterning and curing; **f** Au and Pt black electroplating; **g** physically detach the device from the substrate. *Arrows* indicate the diffusion holes

We designed and fabricated the MEAs with the distribution of diffusion holes among the high-density electrode array such that the retina tissue could be under adequate oxygen supply during the in vitro study. Perforated MEA samples were fabricated on the silicon, as illustrated in Fig. 3. First, wafers were deposited using a thin polymer for releasing the device at the final step. The first layer of a positive photoresist polyimide (Durimide 7320, Fujifilm) with a thickness of 8 μm was then deposited using a spin coater (Fig. 3b). The polyimide was patterned and baked at 350 °C in a nitrogen atmosphere for 3 h (Fig. 3c). A titanium layer with a 40-nm thickness served as the adhesion layer and an Au layer with 300-nm thickness served as the seed layer for deposition (Fig. 3d). The second layer of a negative photoresist polyimide (PW1500, Toray) was used as a cover layer (Fig. 3e). The exposed microelectrodes were electrodeposited platinum black (Fig. 3f). The platinum black coatings were electroplated in chloroplatinic acid solution placed in ultrasonic bath [35].

### Experimental validation

**Fig. 4:**
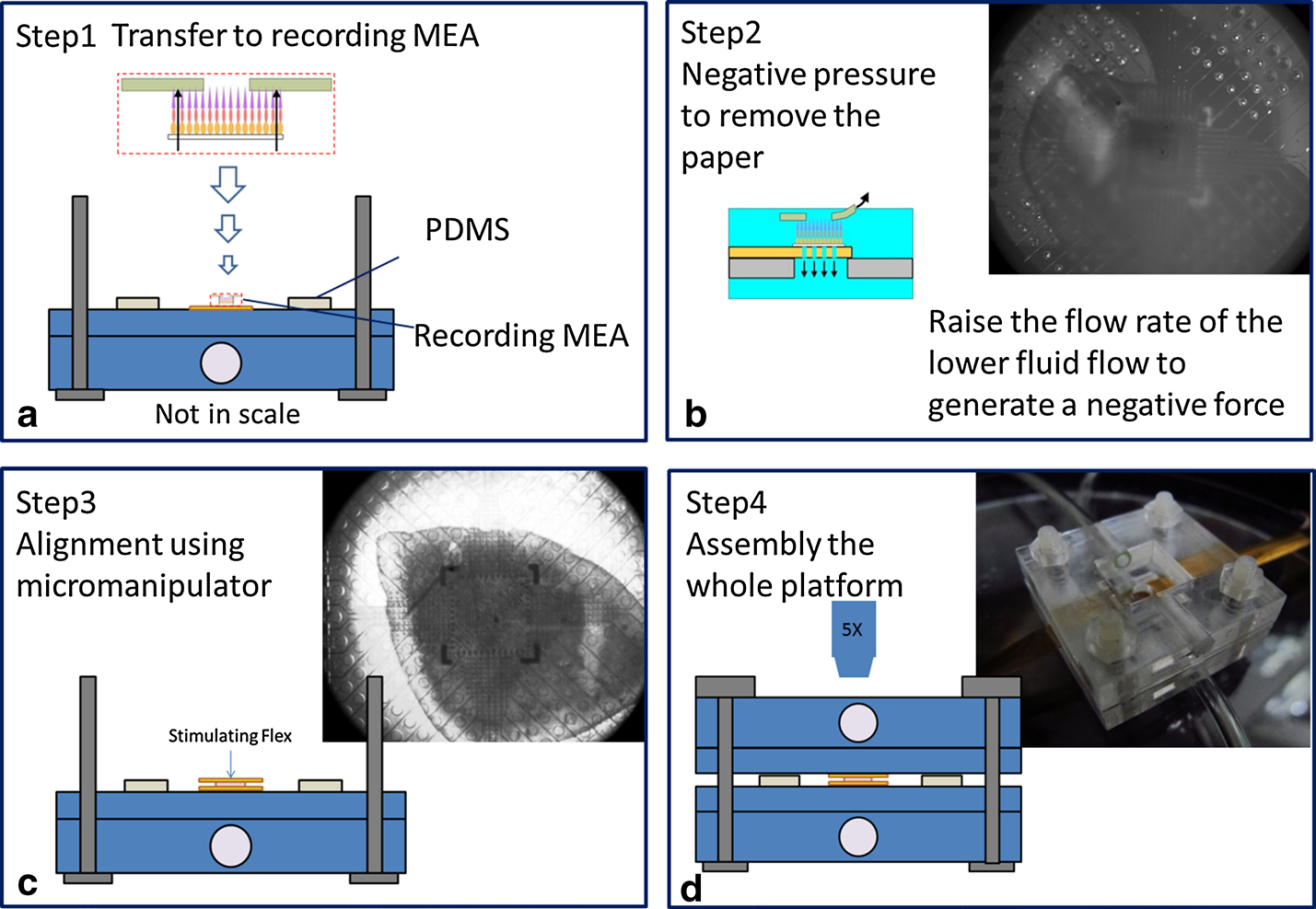
Stepwise assembly of MTM platform. The paper was flipped and the RGC layer was facing down and contacted with the rMEA as the *Step 1*. In *Step* *2*, the flow rate of the lower fluid flow was raised to generate a negative pressure that could hold the retinal tissue on the rMEA surface. In *Step 3*, the sMEA was then added on *top* of the photoreceptor cells and aligned using a micromanipulator. In *Step 4*, the upper perfusion chamber was put in place and the assembled MTM platform was placed under the microscope in the electrophysiological rig

Male C57BL/6 mice of 4–6 weeks old were used in this study. All animal experiments were approved by the National Taiwan University College of Medicine and College of Public Health Institutional Animal Care and Use Committee. The dark-adapted mice were sacrificed in a darkroom. The eyes were hemisected, and lens and vitreous were removed, preserving the visual streak in an artificial cerebrospinal fluid (ACSF, in mM: 126 NaCl; 2.5 KCl; 1.25 NaH_2_PO_4_; 1.3 MgCl_2_; 26 NaHCO_3_; 2.5 CaCl_2_; 10 glucose, pH 7.5 ± 0.2) with carbogen- bubbled (95 % O_2_–5 % CO_2_) at room temperature. Visual streak was cut into three pieces and mounted flat on filter paper (Millipore) with a 1 mm-diameter hole. The paper was flipped and the RGC layer was facing down and contacted with the recording MEA (rMEA) as illustrated in the Step 1 of Fig. 4. The rMEA was coated with cellulose nitrate to enhance its adherence with the RGCs. Various thicknesses of Polydimethylsiloxane (PDMS, elastic materials) were selected as the spacer in the sandwich structure to avoid the intrinsic stress occurred in the retinal tissue. The filter paper was then slowly peeled off and the retina was left on the rMEA. Meanwhile, the flow rate of the lower fluid flow was raised to generate a negative pressure that could hold the retinal tissue on the rMEA surface (Step 2 in Fig. 4). The stimulation MEA (sMEA) was then added on top of the photoreceptor cells and aligned using a micromanipulator (MPC-200, Sutter instrument, USA) (Step 3 in Fig. 4). Finally, the upper perfusion chamber was put in place and the assembled MTM platform was placed under the microscope in the electrophysiological rig (Step 4 in Fig. 4). Here, the top and bottom microelectrode arrays use the same design.

The retina slice between the two MEAs was perfused with carbogen-bubbled ACSF (30 °C, 95 % oxygen bubbles) at a constant volume rate, 100 ml/h. The retina slice was allowed to settle and adapt to the new environment (negative pressure, change in temperature) for 25 min before recording.

**Fig. 5:**
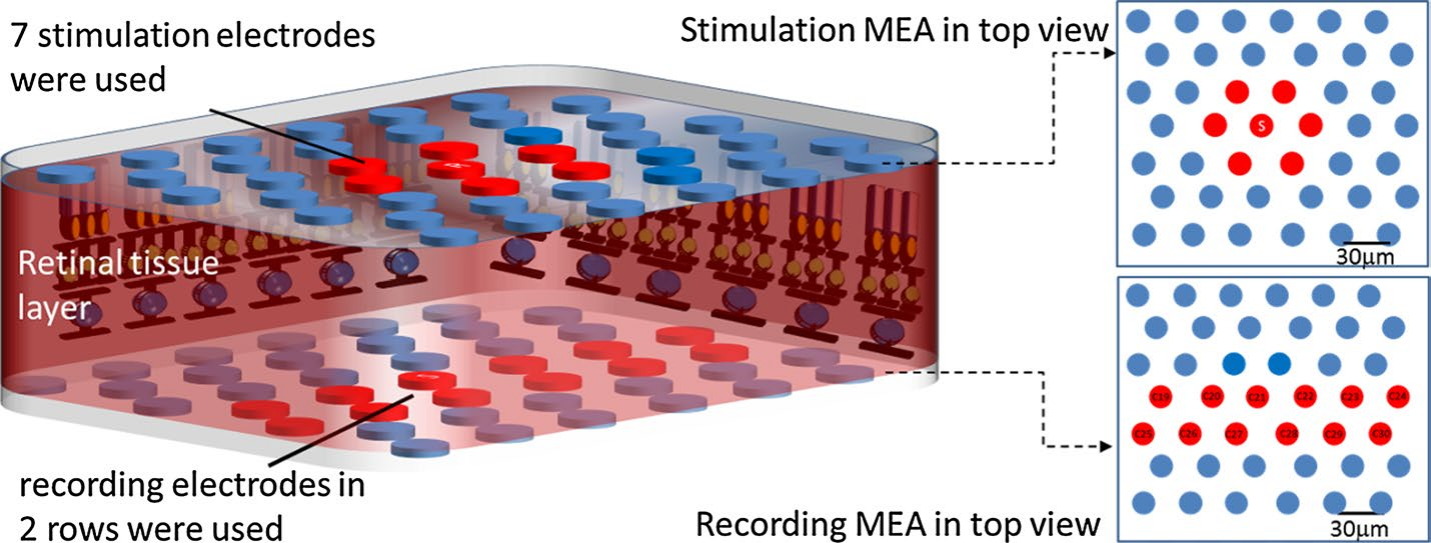
Illustration of sMEA and rMEA. 7 Stimulation electrodes within a hexagon area were used to activate the retina. Sixteen recording aligned in two rows were used to collect the signals

The stimulating electrode was designed as small as 11.5 m in diameter. Pt black was electroplated on the surface to provide a high charge injection capacity. The charge injection capacity of each electrode is limited by the electrode material used and the size of electrode. In this implementation, a single Pt electrode used for stimulation does not have adequate charge injection within the water window to achieve RGC spiking; however, the RGC spiking was achieved within the water window when 7 (1 center and six surrounding) electrodes were simultaneously used with the same electrical stimulation waveform shown as Fig. 5. In this study, whole cell patch method was used measure the threshold voltage of RGCs.

Then the stimulation signals of two-fold of the threshold voltage (1.8 V) were created by programmable waveform generators (PXI6723, National Instruments, USA) in constant voltage mode (1.5 Hz with 5 ms duration) were delivered to the sMEA by 42 multi-channels. The data acquisition system consisted of a 16-channel pre-amplifier (μPA16, Multi Channel Systems MCS Gmbh, BW Germany), backend amplifier, data acquisition card (PXI6289, National Instruments, USA) and user interface (Labview software, National Instruments, USA).

### Data analysis

Seven stimulation electrodes within a hexagon area about 60 × 60 μm^2^ were used to activate the retina from the photoreceptor layer simultaneously. Sixteen electrodes aligned in two rows were used to collect RGC response (n = 5 retina slices). To analyze the raw data, an off-line spike sorting software (Clampfit 10, Molecular Devices, LLC., USA) was used to detect and quantify the spikes. Numerical values were given as mean ± standard error of the mean.

## Results

### Design and simulation of MTM platform

For an ideal MTM platform, the retina tissue clipped by two MEAs should be supplied with sufficient oxygen that the proper physiological condition could be maintained. Furthermore, the density of microelectrodes on the MEAs should be great that the precise point-to-point stimulation-recording relationship could profit the decoding of retinal neural circuits. However, these two concerns are somewhat contradictory, although the problem of oxygen supply might be overcome by the opening of holes in MEAs that could also reduce the density of microelectrodes on the MEAs. Since the central region of the MEA is crowded with microelectrodes and metal wires, the hole size and open area ratio (OAR) in the central area should be small. Accordingly, different sizes of diffusion holes were designed in different regions. In the center, middle and outer regions of the MEA, the diameters of diffusion holes were 10, 50 and 100 μm with 10, 20 and 20 % OAR, respectively.

**Fig. 6:**
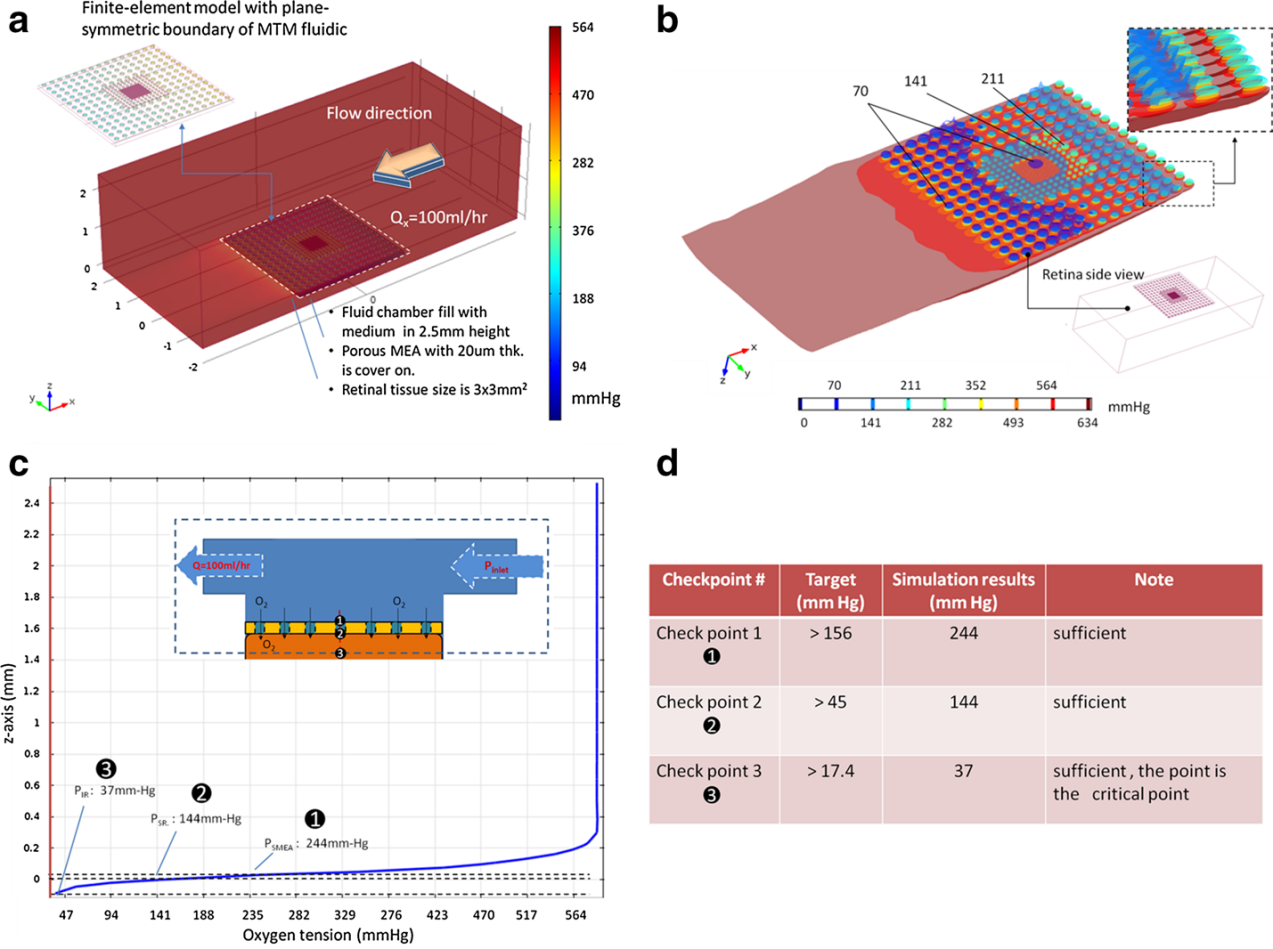
Results of oxygen tension distribution in the fluid chamber. **a** Plane-symmetry model was used to simplify the FEM calculation, and the simulated oxygen tension distribution in the perfusion chamber is shown as the volume plot. **b** Nine iso-surfaces of oxygen tension were used to describe oxygen distribution in the MTM structure. **c** Oxygen tension of the central MTM platform is plotted along the z axis and three check points are also *marked*. **d** Comparison of target values and simulation results

The perfusion flow rate was set to be 100 ml/h and the concentration of oxygen in the inlet of the perfusion system was measured as 18 mg/l using a dissolved oxygen meter. According to the Henry Law (6), the oxygen tension in the inlet (P_inlet_) was 583 mmHg. Based on these parameters, oxygen tension distribution in the fluid was simulated using FEM (Fig. 6a) and nine color-coded isosurfaces from 0 to 634 mmHg were used to visualize the oxygen tension distribution in the MTM structure (Fig. 6b). The results showed the oxygen tension is almost constant at distance to MEA from 0.3 to 2.5 mm. But oxygen level decreased rapidly when the distance was smaller than 0.3 mm (close to the surface of MEA). The oxygen tensions of the three check points were simulated to be 244 mmHg (P_SMEA_) in check point 1 (the outer surface the MEA), 144 mmHg (P_SR_) in check point 2 (the surface between retina and MEA) and 37 mmHg (P_IR_) in check point 3 (the middle of the retina) (Fig. 6c). All these values exceeded the minimum requirements (Fig. 6d), indicating that a retina tissue between the two MEAs could be supplied with sufficient oxygen via the penetrated holes using the perfusion system.

### Fabrication and assembly of MTM platform

**Fig. 7:**
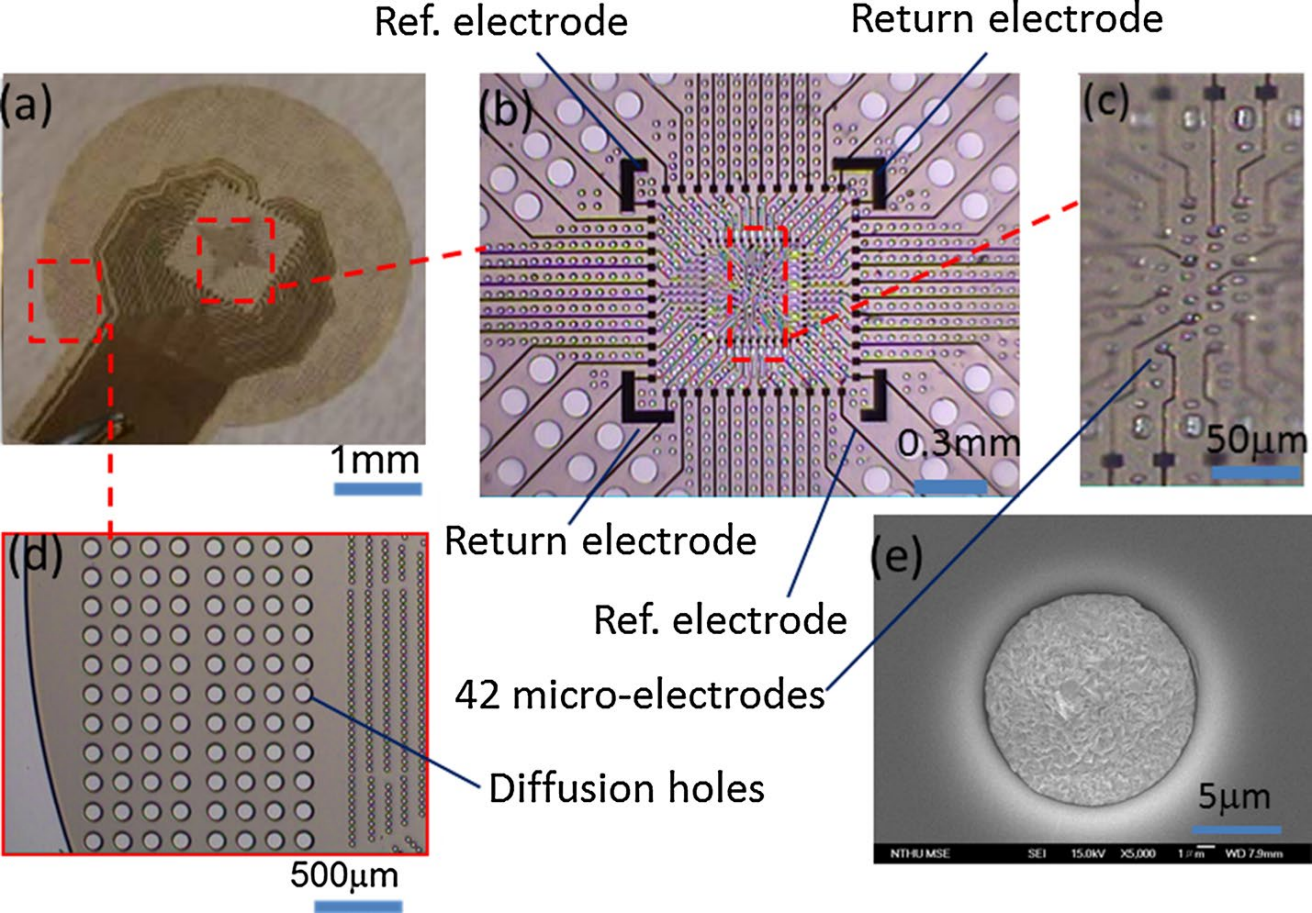
Optical microscope picture of the MEA device. **a** Picture of the head of the MEA. **b** Enlarged picture of the head of the MEA. **c** The central region of the MEA, note the microelectrodes and diffusion holes. **d** The outer region of the MEA. **e** SEM photo of the microelectrode (×5000). The diameter is about 11.5 μm

The fabricated MEA (Fig. 7a) had a total thickness of 20 μm, microelectrodes were located in the central region (Fig. 7b) and there were 48 leads which 12 leads in each side going into MEAs, 42 leads were individually designed for 42 MEA, 4 leads were designed for 2 return electrodes, and 2 leads were designed for 2 reference electrodes. The sizes of the perforation holes were 10, 50 and 100 μm in the center (Fig. 7c), middle (Fig. 7b) and outer (Fig. 7d) regions, respectively. The diameter of individual microelectrode was measured as 11.5 μm using the SEM micrograph (Fig. 7e). The impedance of the microelectrode was 50 kΩ and the double-layer capacitance was 2 nF.

**Fig. 8:**
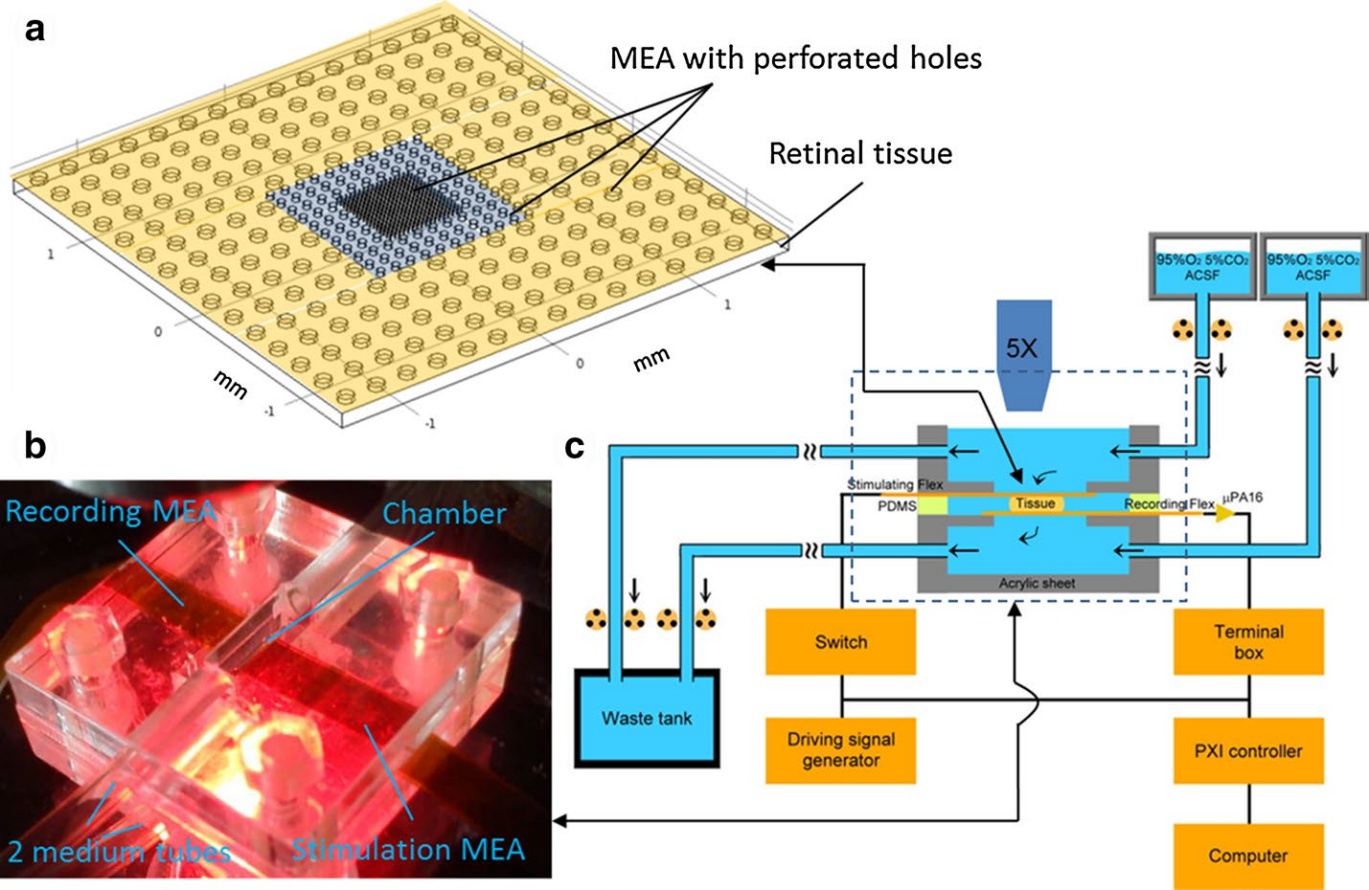
Illustration of MTM platform. **a** The perfusion holes in the MEA surface. In the center (*gray*), middle (*blue*) and outer (*yellow*) regions of the MEA, the diameters of diffusion holes were 10, 50 and 100 μm with 10, 20 and 20 % OAR, respectively. **b** The acrylic perfusion chamber under the microscope. **c** The experimental setup of MTM platform. The perfusion system and the directions of the fluid flow are labeled

The porous high-density MEAs linked with stimulation or recording devices within a perfusion system were manufactured and the MTM platform was assembled with a retina slice inside as illustrated in Fig. 8. The MTM platform consisted of two perfusion paths (Fig. 8c): an upper path supplies the tissue with oxygen via diffusion holes of the stimulation-MEA (sMEA); the lower path supplies the tissue with oxygen via diffusion holes of the recording MEA (rMEA). The differential flow rates produced a negative pressure to pull the retina toward the rMEA and hold it in place.

### Experimental validation of MTM platform

**Fig. 9:**
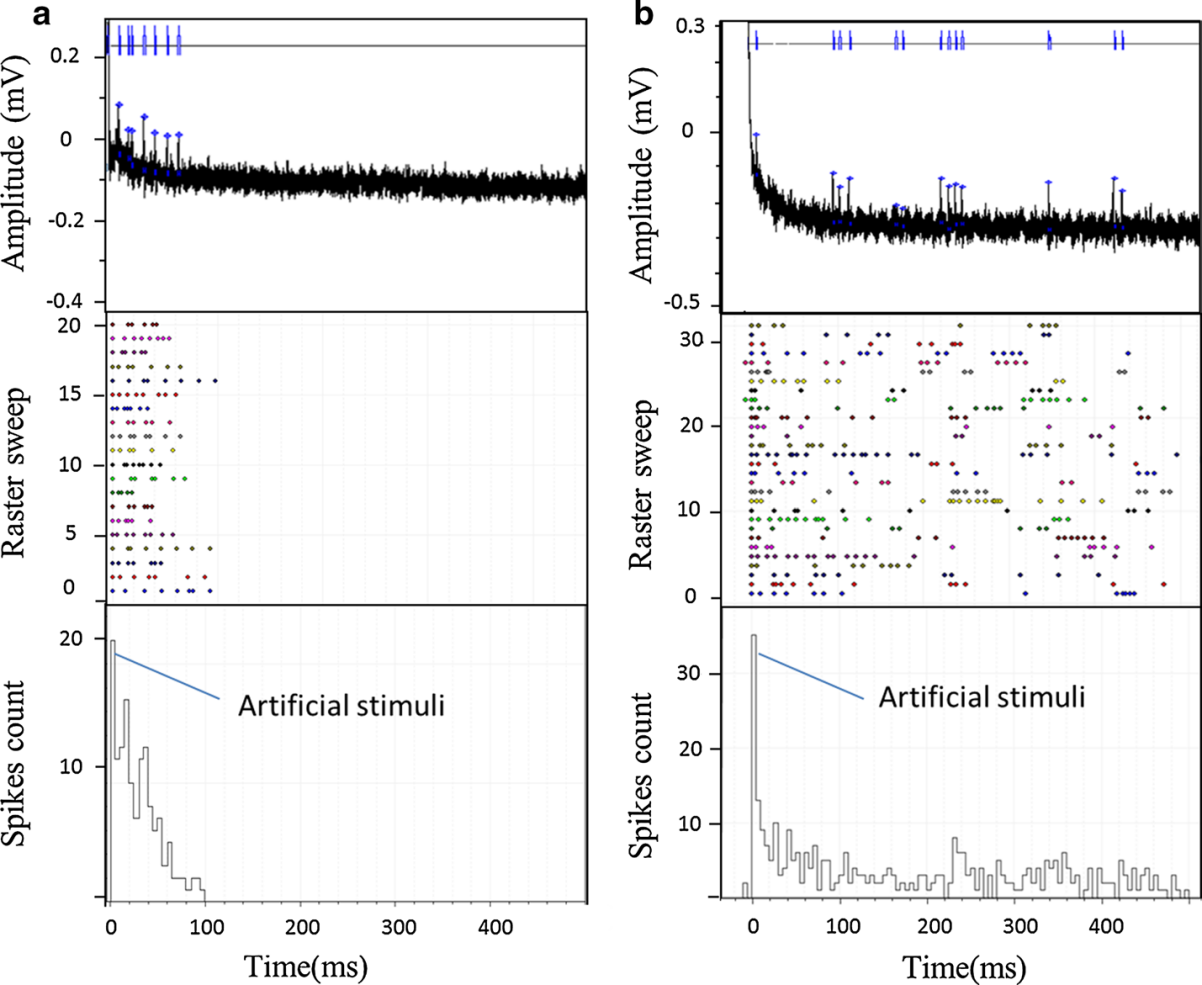
Experimental design of RGCs response and the results. Type I RGC (**a**) and a Type III RGC (**b**) to the electrically artificial stimulation. The histograms show the responses of Type I RGC to 20 repeated stimulations and Type III RGC to 32 repeated stimulations. For stimulation at 1.5 Hz, the bin width was 10 ms. The raster plots above the histograms show the spike activity of each stimulation

After a habitation period of 20–25 min, the responses of RGCs following subretinal electrical stimulation were examined. A pulse train stimulation from the sMEA in voltage mode with a 5 ms pulse width at 1.5 Hz repetition rate in 7 stimulation electrodes within a hexagon area about 60 × 60 μm^2^ were used to activate the retina from the photoreceptor layer. Sixteen electrodes aligned in two rows were used to collect RGC responses (Fig. 5). During recording, different types of RGC responses were noticed (Fig. 9a, b) according to the properties suggested by Jensen and Rizzo (2008) [10]. The pattern of RGC spikes in Fig. 9a resembled to the type I response in which a single burst of spikes within 20 ms is elicited after subretina electrical stimulus [3, 10]. The pattern of RGC spikes in Fig. 9b was similar to the type III cells, which produce two and occasionally three bursts of spikes following subretina stimulation [3, 10].

**Fig. 10:**
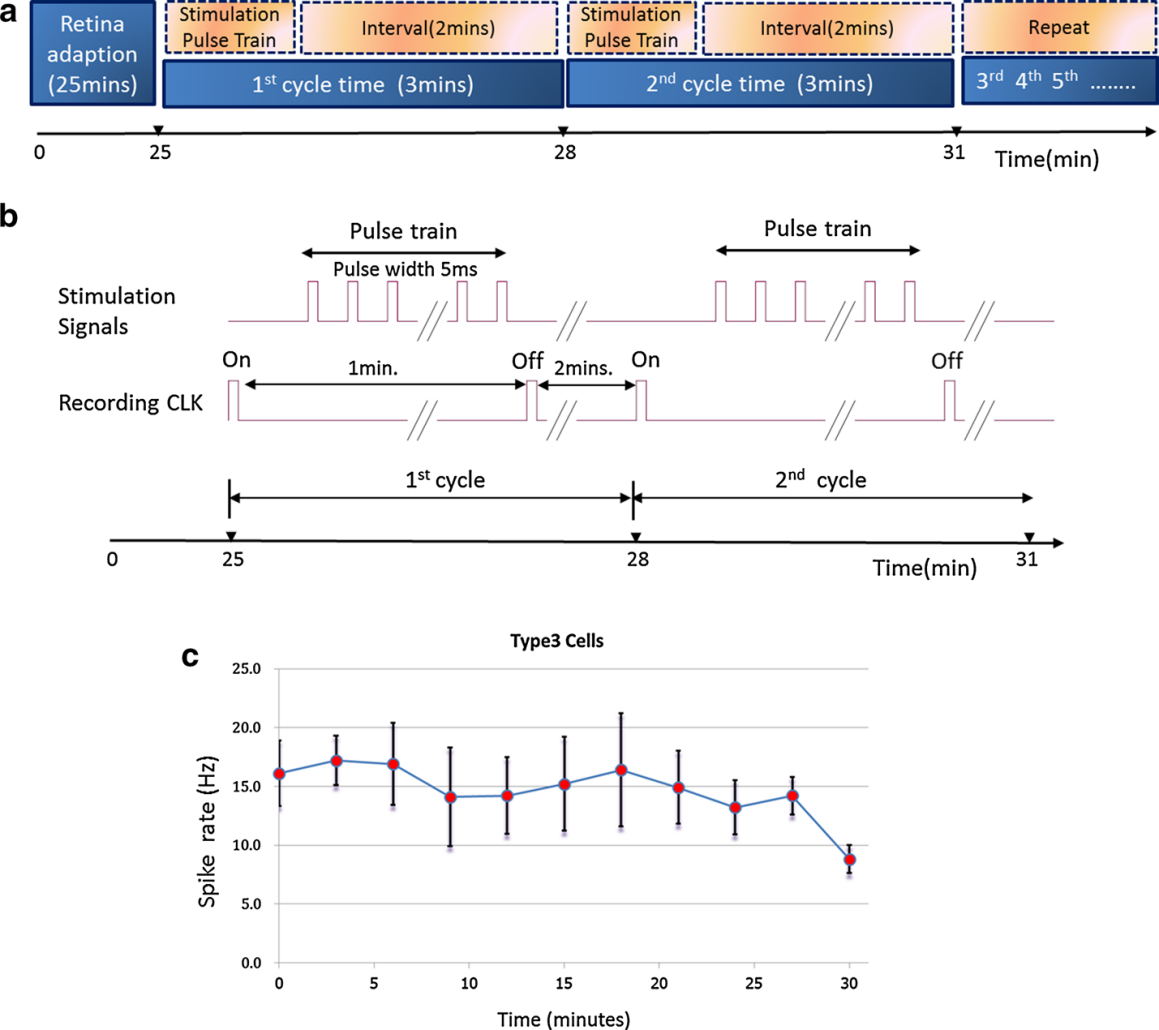
Experimental design of RGC response against time and the results. **a** Schematic diagram of experimental design. **b** Stimulations were delivered every 3 min with 1 min pulse train and 2 min interval. Stimulation signals and time clock control (CLK) of recording signals are indicated. **c** The spike rate of a type III RGC during recording. The first 25 min was the habitation period

We then continuously recorded the RGC responses following repetitive stimulation (Fig. 10). Stimulations were delivered every 3 min (Fig. 10a) with 1 min pulse train and 2 min interval (Fig. 10b). Under the MTM platform, RGCs continuously displayed action potential spikes following subretina stimulation for a certain period of time. For example, a type III RGC exhibited a firing rate about 15 Hz following electrical stimulation redelivered from sMEA (Fig. 10c). The firing rate remained constant between 25 and 55 min before dramatically declined, indicating that within certain period of time (e.g. 30 min after habituation), the retina condition was kept by sufficient oxygen supply via the perfusion holes in the MEAs provided by the double perfusion system. Type I RGC has similar response with the initial spike rate at ~5 Hz.

## Discussion

**Fig. 11:**
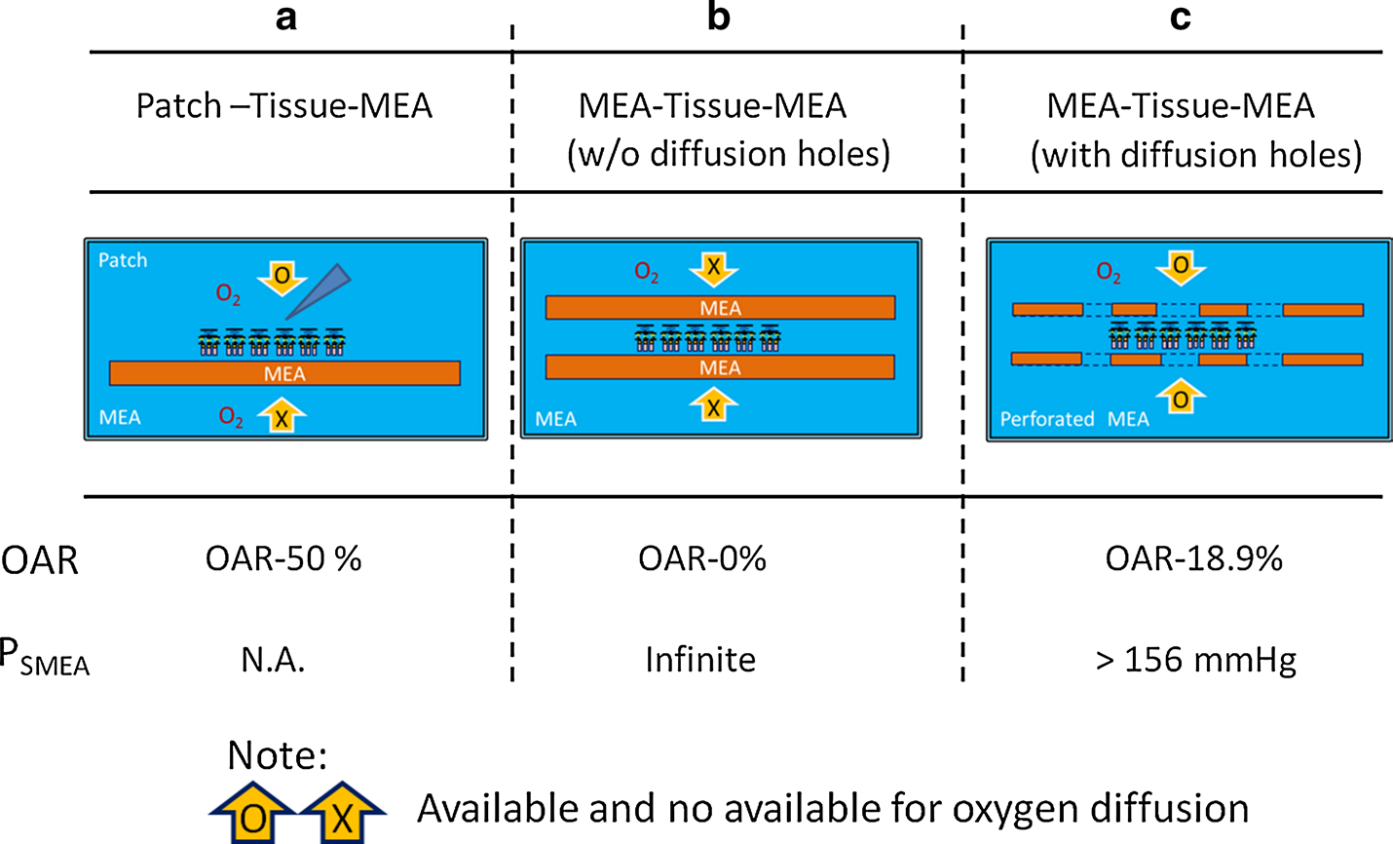
Comparisons of oxygen diffusion with OAR in 3 different structures. **a** Patch-Tissue-MEA, **b** MEA-Tissue-MEA and **c** MEA-Tissue-MEA

In order to understand the retinal information processes, MEAs are commonly used in various in vitro models (Fig. 11). In some studies, MEAs are used to stimulate photoreceptors, the RGC responses are collected using whole-cell patch clamp recording (Fig. 11a). In such studies, the identity and receptive field of RGCs can be characterized. However, it is difficult to simultaneously record multiple RGCs at the same time. The use of small-diameter high-density MEA to record RGC responses may solve this issue, especially the signals obtained from different microelectrodes are separately analyzed. The sMEA is used to stimulate the retinal circuit from the various photoreceptors and the responses of multiple RGCs are recorded by the rMEA. In this MTM configuration, both MEAs should be closely contacted with retina tissue that the stimulating and recorded signals are faithfully delivered and collected, respectively. However, in such scenario, the supply of oxygen to the clipped retina may be significantly hindered (Fig. 11b). The opening of perfusion holes (Fig. 11c) is our way to resolve the problem. In fact, to fabricate an ideal MTM platform, three aims should be achieved at the same time. First, the diameter of microelectrodes should be small and the density should be great. Second, the supply of oxygen should be secured. Finally, the distances between retina and MEAs should be minimum.

The diameter of Pt black microelectrodes was 11.5 μm and the distance between electrodes was 30 μm, that the spatial resolution of the MEAs was estimated to be visual acuity (VA) 0.16, better than the level of legally blind VA 0.1 (American Medical Association, AMDA). Besides, Pt black has excellent electrochemical property such as low impedance. Together, the material, diameter and density of the MEA make it suitable for retinal neural circuit study and retinal prosthesis. The opening of perfusion holes was also included in the fabrication process. In the central region of the MEA, the oxygen supply through the 10 μm holes with 10 % OAR from the dual perfusion paths were simulated to be sufficient. This, to the best of our knowledge, is the first attempt to simulate the oxygen tension within the clipped retina tissue. The perfusion system not only provide the flow of oxygenated fluid for gas supply but also a negative pressure to pull the retina close to the MEAs, that the noise signals were reduced during recording.

Generally using whole-cell recording technique, the condition of a RGC can be held for up to 1 h. The recording time can be even prolonged using loose patch or extracellular recording paradigm. Using our MTM platform, the activity of RGCs can be kept for about half an hour before its decline. It is not satisfactory. We are still fine-turning the preparation of retina slices, constituents of perfusion fluid and flow rate. Sufficient recording time is important for large-scaled multiple stimulation and recording paradigm to decode the retinal information processes.

Using the small-diameter high density rMEA, different types of RGC were characterized based on the electrically evoked responses [3, 10]. It should be noticed that such RGC responses were first identified using whole-cell patch clamp recording technique [10]. Our recording and spike shorting techniques make it possible to analyze the RGC responses close to the single-cell resolution. The goal of simultaneous recording of multiple RGCs might be accomplished in the near feature, which could make substantial contribution to retinal circuit research.

For our present MTM platform and stimulation/recording system, there is still room for improvement, especially in reducing the signal-to-noise ratio (SNR) and prolonging the recording time. Out of 16 recording electrodes, RGC responses can only be analyzed from those that had higher SNR. The numbers were only 6 electrodes dependent upon the condition of the retina. There are several possible ways to increase the SNR. For examples, by increasing the perfusion flow rate that negative pressure is raised to draw near the MEAs and retina. Alternatively, developing lower-impedance electrodes may be another route to increase SNR. It is likely that the retina tissue deteriorated during the in vitro operation and limited the recording of neural activity to ~30 min. Reducing the preparation-and-positioning time of the in vitro operation, and using a milder negative pressure to hold the tissue in the future may prolong the tissue survival time.

## Conclusion

MTM sandwich structure consists of two perforated microelectrode arrays, for stimulation and recording, respectively, and a clipped retinal tissue in between. It is the most efficient platform to study the retinal neural circuit. The material and arrangement of high density microelectrodes with porous design make this MEA appropriate for sub-retina prosthesis. Finding ways to prolong the recording time and reduce the signal-to-noise ratio are important to improve our MTM prototype.

## Abbreviations

MEA: Microelectrode array
rMEA: Recording MEA
sMEA: Stimulation MEA
MTM: MEA-tissue-MEA
RGCs: Retinal ganglion cells
AMD: Age-related macular degeneration
RP: Retinitis pigmentosa
PO2: Oxygen tension
OAR: Open area ratio
FEM: Finite element method
VA: Visual acuity
SNR: Signal to noise ratio
